# Neutrophilic granulocyte percentage is associated with anxiety in Chinese hospitalized heart failure patients

**DOI:** 10.1186/s12872-022-02940-y

**Published:** 2022-11-20

**Authors:** Qian Ma, Feng-bo Zhang, En-sheng Yao, Shuo Pan

**Affiliations:** 1 First Department of Cardiology, First Affiliated Hospital, School of Medicine, Shihezi University, Shihezi, Xinjiang People’s Republic of China; 2 The Clinical Laboratory Medical Center, The First Affiliated Hospital of Xinjiang Medical University, Urumqi, Xinjiang People’s Republic of China; 3 Department of Neurology, First Affiliated Hospital, School of Medicine, Shihezi University, Shihezi, Xinjiang People’s Republic of China; 4 Cardiovascular Department, People’s Hospital of Shaanxi Province, Xi’an, Shaanxi People’s Republic of China

**Keywords:** Neutrophilic granulocyte percentage, Anxiety, Hospitalized heart failure, Chinese

## Abstract

**Background:**

In patients with heart failure, anxiety disorder is common and associated with adverse prognosis. This study intended to find more confounding factors of Chinese heart failure patients.

**Methods:**

We enrolled 284 hospitalized heart failure patients, whose New York Heart Association (NYHA) classed as II-IV and left ventricular ejection fraction (LVEF) ≤ 45%. All the patients were scaled in Hamilton Rating Scale for Anxiety (14-items) (HAM-A14). Ordinal logistic regression analysis was performed to examine the association of correlated factors with anxiety disorder.

**Results:**

There were 184 patients had anxiety accounting for 64.8% of all 284 hospitalized heart failure patients. The neutrophilic granulocyte percentage, urea nitrogen, total bilirubin and brain natriuretic peptide were positively associated with HAM-A14 score, meanwhile, the hemoglobin, red blood cells counts, albumin and LVEF were negatively associated with HAM-A14 score (All *P* < 0.05). After the adjustments of sex, hemoglobin, urea nitrogen, total bilirubin, albumin and brain natriuretic peptide, the neutrophilic granulocyte percentage was significantly associated with anxiety (OR = 43.265, *P* = 0.012). The neutrophilic granulocyte percentage was 0.616 ± 0.111, 0.640 ± 0.102, 0.681 ± 0.106 and 0.683 ± 0.113 in heart failure patients with no anxiety, possible anxiety, confirmed anxiety and obvious anxiety, respectively.

**Conclusions:**

Neutrophilic granulocyte percentage as well as the traditional risk factors such as sex, urea nitrogen and brain natriuretic peptide is associated with anxiety in hospitalized heart failure patients.

## Introduction

Heart failure is highly prevalent around the globe, the estimated prevalence will increase by 46% till 2030, resulting in 8 million adults suffering from heart failure of the US [1]. The situation is the same in developing countries, the estimated heart failure cost of China was $5.42 billion every year, and accounting for 5% of total health care costs and the cost is still increasing dramatically in recent years [2].

It is reported that prevalence of anxiety in heart failure patients ranged 11% to 70% in different to the literatures [3–8]. Severe anxiety is also associated with the incident cardiovascular diseases [9, 10] and also with increased mortality risk of heart failure patients particularly when LVEF is severely decreased [11].

The literature focusing on the risk factors of anxiety in heart failure patients was very limited based on Chinese population. In this present study, we intended to find more confounding factors of anxiety in Chinese heart failure patients with both left ventricular ejection fraction (LVEF) ≤ 45% and New York Heart Association (NYHA) class II-IV.

## Methods

### Study design, setting and participants

The present study was performed from January of 2019 to January of 2021 and retrospectively analyzed in First Affiliated Hospital, School of Medicine, Shihezi University. The hospitalized Chinese patients with heart failure were recruited in this study, all the recruited heart failure patients should have both left ventricular ejection fraction (LVEF) ≤ 45% and New York Heart Association (NYHA) class II-IV within the previous 6 months [12, 13]. The anxiety is defined by diagnostic and statistical manual of mental disorders fifth edition (DSM-5).All patients were decompensated chronic heart failure patients, and the patients with acute heart failure, acute coronary syndrome, fever, current use of antipsychotic or anti-anxiety medications, life-threatening comorbidity, suicidal ideation, severe personality disorder, bipolar disorder, psychoses, alcohol or drug dependence and cognitive impairment were excluded in the study.

### Anxiety assessments

Hamilton Rating Scale for Anxiety (14-items) (HAM-A_14_) was used to evaluate the anxiety symptoms in the heart failure patients within 3 days after admission. Hamilton Rating Scale for Anxiety (14-items) contained 14 items for evaluating the anxiety levels. Each item has one question on the anxiety status, the answer to each question is scored from 0 to 4 (none (0), mild (1), moderate (2), severe (3), very severe (4)). The total score was calculated. HAM-A_14_ scored of 0–7 points was defined as no anxiety status, HAM-A_14_ scored of 8–14 points was defined as possible anxiety status, HAM-A_14_ scored of 15–21 points was defined as confirmed anxiety status, HAM-A_14_ scored of 22–29 points was defined as obvious anxiety status, HAM-A_14_ scored of > 29 points was defined as severe anxiety status [14, 15]. The questionnaire of HAM-A_14_ was conducted by trained physicians on the first day after admission. Two physicians in our department were sent to psychology department for the training of talking, observing and interpreting skill of HAM-A_14_ for a week. HAM-A_14_ scores were measured by two physicians independently for each patient, the average score of the two scores calculated by the two physicians were used as the final score. If the two scores were diverted over 2 points, the psychologist in psychology department would repeat the test and calculate the final score.

### Demographic and clinical data

Demographic data, diagnosis and NYHA classification were obtained from the medical records in each patient. Monthly family income, habitant area and education background were documented when the patients were admitted. BMI was calculated as body weight/ height^2^ (kg/m^2^). Smoking index was calculated as cigarettes number per day multiplied with smoking years.

### Blood samples and transthoracic echocardiography

On the next day of admission, two tubes of cubital fasting vein blood were collected and sent to the clinical laboratory test. The test results were recorded, in which EDTA-k2 anticoagulant tube whole blood was used for blood routine, hemoglobin A1c (HbA1c), BNP testing, and biochemical examination with heparin anticoagulant tube plasma included: hepatic function, renal function, blood lipid. Transthoracic echocardiography was conducted and the LVEF was documented for further analysis.

### Definition of comorbidities

Coronary artery disease was defined as over 50% diameter stenosis in at least one artery, which is confirmed using coronary angiography or coronary computed tomographic angiography (CTA).

Hypertension was defined as systolic blood pressure ≥ 140 mm Hg, or diastolic blood pressure ≥ 90 mm Hg, or antihypertensive medication, or previous diagnosed hypertension.

Atrial fibrillation was defined as disappearance of P waves and diverted R-R intervals according to electrocardiogram (ECG).

Dilated cardiomyopathy (DCM) is characterized by left ventricular (LV) systolic dysfunction and LV enlargement, in the absence of abnormal loading conditions such as hypertension, valvular disease, or coronary artery disease (CAD) that could explain the myocardial abnormality. The presence of the disorder is defined by an LV end-diastolic diameter (LVEDD) greater than 2 standard deviations (SD) of the predicted values and LV fractional shortening < 25% or an LV ejection fraction (EF) < 45% [16–18].

Diabetes was defined as fasting plasma glucose over 7.0 mmol/L, or random plasma glucose ≥ 11.1 mmol/L, or positive oral glucose tolerance test (OGTT), or insulin or oral hypoglycemic drug, or diabetes history.

### Statistical analysis

The statistical analysis was conducted using SPSS version 16.0 for Windows (SPSS Inc., Chicago, IL, USA). All continuous variables were tested for normal distribution using Kolmogorov – Smirnov normal distribution test. The normally distributed variables were expressed as mean ± standard deviations and the differences were calculated using the student *t*-test. The non-normally distributed variables were expressed as three quartiles (Q25; Q50; Q75) and compared with Mann–Whitney *U* test. Categorical variables were expressed as proportions and the differences were analyzed using chi-square test or fisher exact test. Pearson (normally distributed variables) and Spearman (non-normally distributed variables) correlation analysis were conducted to determine the correlation between HAM-A_14_ score and each clinical and laboratory factor. All baseline characteristics in heart failure patients were included as univariate analysis variables, and those factors with the statistically significant presence (*P* < 0.1) in anxiety were selected as variables in the multivariate analysis. Logistic regression model was established to determine the association with the neutrophilic granulocyte percentage and anxiety after the adjustment of confounding factors. The neutrophilic granulocyte percentage of each anxiety level was expressed as mean ± standard deviations and compared using one way ANOVA. Statistical significance was established at *P* < 0.05.

## Results

The baseline characteristics of hospitalized heart failure patients with and without anxiety were presented in Table 1. And 284 hospitalized patients with heart failure were enrolled in the present study, among the enrolled 284 hospitalized patients, 174 (61.3%) patients were male and 110 (38.7%) patients were female. The average age was 68.49 ± 12.16 years. There were 184 patients had anxiety accounting for 64.8% of all 284 hospitalized heart failure patients. Among the 184 patients with anxiety, 112 patients (39.4%) had possible anxiety, 77 patients (27.6%) had confirmed anxiety and 13 patients (4.6%) had obvious anxiety, no patient had severe anxiety. 28.2% of men had anxiety while 46.4% of women had anxiety, the distribution of anxiety between men and women was statistically significant (*P* = 0.002). Most of the patients that had anxiety (91.0%) were classified as NYHA classification III and IV, while only 70.1% patients without anxiety were classified as NYHA classification III and IV (*P* < 0.001). The distribution of monthly family income, habitant area, education background, coronary artery disease, hypertension, atrial fibrillation, dilated cardiomyopathy and diabetes mellitus showed no difference in patients with or without anxiety. The patients without anxiety showed higher hemoglobin, triglyceride, HDL-C, apolipoprotein A, albumin and LVEF while the patients with anxiety showed higher NEUT%, BUN, creatinine, TBIL, BNP and HAM-A_14_ score (*P* < 0.05). The age, BMI, smoking index, RBC counts, platelet counts, plateletcrit, MPV, PDW, WBC counts, TC, LDL-C, Apolipoprotein B, FG and HbA1c showed no difference between the patients with or without anxiety.

**Table 1:** Baseline characteristics of Chinese hospitalized heart failure patients with and without anxiety

	Without anxiety (*n* = 184)	With anxiety (*n* = 100)	*P* value
**Sex (%)**			0.002*
Men	125(67.9%)	49(49.0%)	
Women	59(32.1%)	51(51.0%)	
**Monthly family income (%)**			0.187
< 3000 yuan*	55(29.9%)	38(38.0%)	
3000–5000 yuan*	80(43.5%)	44(44.0%)	
> 5000 yuan*	49(26.6%)	18(18.0%)	
**Habitant area (%)**			0.110
Rural area	65(35.3%)	55(55.0%)	
Urban area	119(67.4%)	45(45.0%)	
**Education background (%)**			0.190
Illiterate	17(9.2%)	13(13.0%)	
Primary school	27(14.7%)	21(21.0%)	
Middle school	88(47.8%)	40(40.0%)	
High school	40(21.7%)	24(24.0%)	
College or above	12(6.5%)	2(2%)	
**NYHA classification (%)**			< 0.001*
II	55(29.9%)	9(9.0%)	
III	101(54.9%)	51(51.0%)	
IV	28(15.2%)	40(40.0%)	
Coronary artery disease (%)	133(72.3%)	70(70.0%)	0.684
Hypertension (%)	98(53.3%)	60(60.0%)	0.275
Atrial fibrillation (%)	61(33.2%)	34(34.0%)	0.885
Dilated cardiomyopathy (%)	21(11.4%)	13(13.0%)	0.694
Diabetes mellitus (%)	61(33.2%)	30(30.0%)	0.587
Age (years)	60; 70; 77	59; 69; 76	0.593
BMI (kg/m^2^)	22.49; 23.39; 24.46	22.76; 23.53; 24.52	0.202
Smoking index	0; 200; 500	0; 0; 400	0.090
RBC counts (× 10^12^/L)	3.98; 4.37; 4.78	3.79; 4.19; 4.61	0.088
Hemoglobin (g/L)	122; 134; 144	116; 128; 142	0.019*
Platelet counts (× 10^9^/L)	143; 185; 228	131; 179; 220	0.142
Plateletcrit (%)	0.16; 0.19; 0.24	0.15; 0.18; 0.23	0.214
MPV (fL)	10.78 ± 1.68	11.00 ± 1.28	< 0.001*
PDW (fL)	15.10; 16.83; 17.50	14.10; 16.60; 17.27	0.069
WBC counts (× 10^9^/L)	5.38; 6.31; 7.91	5.23; 6.40; 8.13	0.992
NEUT% (%)	0.62 ± 0.10	0.69 ± 0.11	< 0.001*
TC (mmol/L)	3.74 ± 1.13	3.54 ± 1.13	< 0.001*
Triglyceride (mmol/L)	0.91; 1.21; 1.82	0.73; 1.07; 1.55	0.017*
HDL-C (mmol/L)	1.05 ± 0.29	0.98 ± 0.28	< 0.001*
LDL-C (mmol/L)	1.58; 2.07; 2.73	1.43; 1.97; 2.70	0.384
Apolipoprotein A (g/L)	1.19 ± 0.25	1.10 ± 0.27	< 0.001*
Apolipoprotein B (g/L)	0.62; 0.78; 0.97	0.60; 0.81; 0.98	0.957
BUN (mmol/L)	4.50; 5.53; 7.10	5.23; 6.53; 9.17	< 0.001*
Creatinine (umol/L)	60.14; 71.00; 84.37	63.00; 78.00; 98.00	0.009*
TBIL (umol/L)	11.2; 15.1; 20.7	11.8; 17.0; 25.3	0.024*
Albumin (g/L)	39.04 ± 4.48	37.20 ± 4.59	< 0.001*
BNP (pg/mL)	113; 340; 779	652; 1035; 1929	< 0.001*
FG (mmol/L)	5.2; 5.9; 7.0	5.0;5.7; 7.0	0.921
HbA1c (%)	5.5; 6.0; 6.5	5.5; 6; 6.6	0.639
LVEF (%)	36; 41; 44	18; 37; 43	< 0.001*
HAM-A_14_ score	6; 8; 10	16; 18; 19	< 0.001*

Chinese 1 Yuan Renminbi is equivalent to 0.142 US Dollar

*NYHA* New York Heart Association, *BMI* Body Mass Index, *RBC* Red Blood Cells, *MPV* Mean Platelet Volume, *PDW* Platelet Distribution Width, *WBC* White Blood Cells, *NEUT%* Neutrophilic granulocyte percentage, *TC* Total Cholesterol, *HDL-C* High-Density Lipoprotein-Cholesterol, *LDL-C* Low-Density Lipoprotein-Cholesterol, *BUN* Blood Urea Nitrogen, *TBIL* Total Bilirubin, *BNP* Brain Natriuretic Peptide, *FG* Fasting Glucose, *HbA1c* Glycosylated Hemoglobin A1c, *LVEF* Left ventricular ejection fraction, *HAM-A*_*14*_ Hamilton Rating Scale for Anxiety (14 items)

^*^P < 0.05

Pearson and Spearman correlation analysis between HAM-A_14_ score and clinical and laboratory factors in hospitalized heart failure patients were showed in Table 2. The NEUT%, BUN, TBIL and BNP were positively associated with HAM-A_14_ score, meanwhile, the smoking index, hemoglobin, RBC counts, albumin and LVEF were negatively associated with HAM-A_14_ score. The age, BMI, WBC counts, PDW, platelet counts, MPV, plateletcrit, TC, triglyceride, HDL-C, LDL-C, apolipoprotein A, apolipoprotein B, creatinine and fasting glucose revealed no difference between patients with or without anxiety.

**Table 2:** Pearson correlation analysis between HAM-A_14_ score and clinical and laboratory factors in Chinese hospitalized heart failure patients

	*r*	*P* value
Age	-0.008	0.887
BMI	0.079	0.184
Smoking index	-0.145	0.014*
RBC counts	-0.157	0.008*
Hemoglobin	-0.208	< 0.001*
Platelet counts	-0.104	0.079
Plateletcrit	-0.071	0.232
MPV	0.113	0.057
PDW	-0.025	0.680
WBC counts	-0.038	0.521
NEUT%	0.269	< 0.001*
TC	-0.072	0.225
Triglyceride	-0.093	0.120
HDL-C	-0.082	0.170
LDL-C	0.043	0.471
Apolipoprotein A	-0.108	0.069
Apolipoprotein B	-0.013	0.833
BUN	0.223	< 0.001*
Creatinine	0.120	0.043*
TBIL	0.119	0.045*
Albumin	-0.192	0.001*
BNP	0.331	< 0.001*
FG	-0.104	0.350
HbA1c (%)	0.032	0.596
LVEF	-0.186	0.002*

*HAM-A*_*14*_ Hamilton Rating Scale for Anxiety (14 items), *BMI* Body Mass Index, *NYHA* New York Heart Association, *RBC* Red Blood Cells, *MPV* Mean Platelet Volume, *PDW* Platelet Distribution Width, *WBC* White Blood Cells, *NEUT%* Neutrophilic granulocyte percentage, *TC* Total Cholesterol, *HDL-C* High-Density Lipoprotein-Cholesterol, *LDL-C* Low-Density Lipoprotein-Cholesterol, *BUN* Blood Urea Nitrogen, *TBIL* Total Bilirubin, *BNP* Brain Natriuretic, *FG* Fasting Glucose, *HbA1c* Glycosylated Hemoglobin A1c, *LVEF* Left ventricular ejection fraction

^*^P < 0.05

Logistic regression analysis for anxiety were conducted, the neutrophilic granulocyte percentage and confounding factors were put into the logistic regression model (Table 3). Since the significantly HAM-A_14_ correlated continuous variables and significantly different categorical variables were enrolled in the logistic regression to determine the associated factors for anxiety of heart failure patients. Since the hemoglobin and RBC counts, BUN and creatinine, NYHA classification and BNP and LVEF were highly resembled with each other, they can’t be put into the logistic regression simultaneously. Therefore, the hemoglobin, BUN and BNP were selected since they had significantly higher correlation coefficient with HAM-A_14_ score. After the adjustments of sex, smoking index, hemoglobin, BUN, TBIL, albumin and BNP, the NEUT% was significantly associated with anxiety (*OR* = 43.265, *P* = 0.012).

**Table 3:** Logistic regression analysis for HAM-A_14_ determined anxiety using neutrophilic granulocyte percentage and each confounding factor in Chinese hospitalized heart failure patients

	Odd ratios	95% *CI*	*P* value
Sex (women)	5.056	2.243–11.537	< 0.001*
NYHA classification (%)	1.045	0.863–2.164	0.295
Smoking index	1.032	0.916–1.024	0.082
RBC counts (× 1012/L)	1.016	0.954–1.127	0.472
Hemoglobin	1.046	0.952–1.062	0.665
MPV (fL)	2.436	0.855–2.673	0.371
PDW (fL)	1.054	0.781–1.428	0.284
NEUT%	43.265	4.117–837.328	0.012*
TC (mmol/L)	2.663	0.541–3.041	0.125
Triglyceride (mmol/L)	1.098	0.857–1.972	0.651
HDL-C (mmol/L)	1.256	0.861–2.462	0.325
Apolipoprotein A (g/L)	0.931	0.892–1.224	0.164
Creatinine (umol/L)	1.075	0.867–1.243	0.085
BUN	1.207	1.066–1.476	0.004*
TBIL	1.325	0.897–1.145	0.123
Albumin	0.760	0.692–1.236	0.306
BNP	1.012	1.078–1.251	< 0.001*
LVEF (%)	0.987	0.685–1.329	0.263

*HAM-A*_*14*_ Hamilton Rating Scale for Anxiety (14 items), *CI* Confidence Interval, *RBC* Red Blood Corpuscle, *MPV* Mean Platelet Volume, *PDW* Platelet Distribution Width, *TC* Total Cholesterol, *HDL-C* High-density lipoproteincholesterol, *NEUT%* Neutrophilic granulocyte percentage, *BUN* Blood Urea Nitrogen, *TBIL* Total Bilirubin, *BNP* Brain Natriuretic Peptide

^*^P < 0.05

**Fig. 1:**
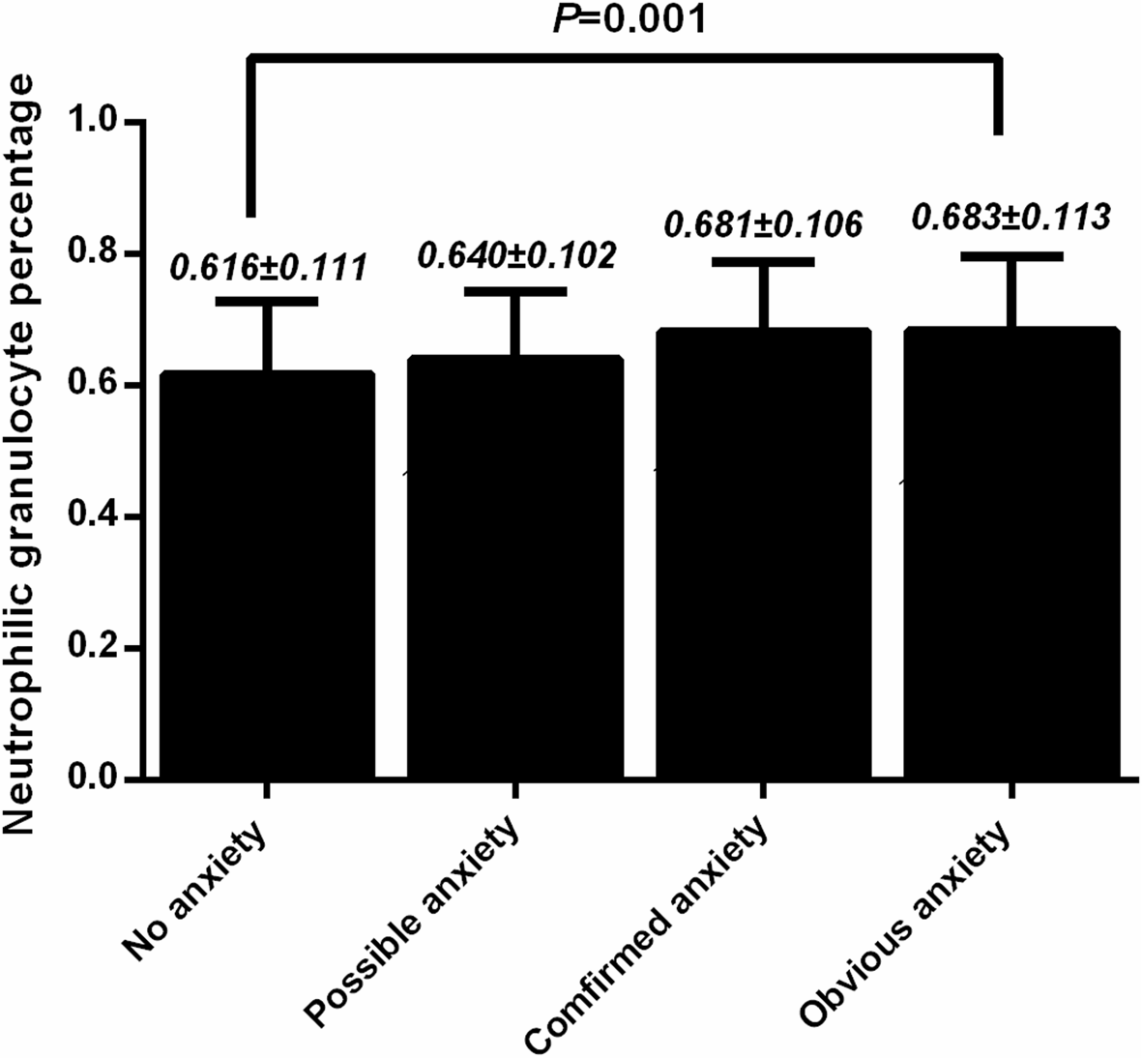
NEUT% in Chinese heart failure patients with no anxiety, possible anxiety, confirmed anxiety and obvious anxiety

The NEUT% in heart failure patients with no anxiety, possible anxiety, confirmed anxiety and obvious anxiety were calculated in Fig. 1. The NEUT% was 0.616 ± 0.111, 0.640 ± 0.102, 0.681 ± 0.106 and 0.683 ± 0.113 in heart failure patients with no anxiety, possible anxiety, confirmed anxiety and obvious anxiety, respectively.

## Discussions

Heart failure is one of the most common cardiovascular diseases, an estimated 26 million adults worldwide are suffering from heart failure [19]. Since the heart failure is the end stage in majority of cardiovascular diseases, so the prevalence of heart failure is still increasing with increase trend of coronary artery disease, myocardiopathy and valvular heart disease [20–22]. Anxiety is quite common among patients with heart failure [23–25]. The prevalence of anxiety in heart failure patients showed 4–5 times increase than that in the general population. In this present study, we have noticed that 184 patients had anxiety accounting for 64.8% of all 284 hospitalized heart failure patients, while previous studies have shown that prevalence of anxiety in heart failure patients ranged from 11 to 70%. The prevalence difference in different studies may be due to different enrollment criteria, heart failure definition and severity of the cases included in each study [26, 27]. Our study has documented relatively higher level of prevalence of anxiety, the reasons may be that the heart failure patients we enrolled were mainly hospitalized patients with moderate or severe symptoms, then the prevalence might be higher than that in other studies which mainly enrolled heart failure outpatients [28].

In this present study, after the adjustments of sex, smoking index, hemoglobin, BUN, TBIL, albumin and BNP, the NEUT% was significantly associated with anxiety (*OR* = 43.265, *P* = 0.012). The *OR* values reached 43.265 indicating that the NEUT% has significant effect on anxiety level in Chinese heart failure patients. The reason for the association is not entirely clear, the possible mechanisms may be as follows. Studies have showed that acute inflammation increases anxiety even in healthy subjects [29], and inflammation is increasingly interpreted as a cofactor in the pathophysiological processes of anxiety [30, 31]. A recent meta-analysis has included 41 studies comparing the inflammation status in anxiety disorders population and healthy controls, the results demonstrated a significant overall difference in inflammation factors such as pro-inflammatory cytokines, interleukin-1β, IL-6, and tumor necrosis factor [32]. The neutrophilic granulocytes and other inflammation factors would be activated when responding to inflammation exposure stimulated by heart failure, the activated inflammation status may then contribute to the aggravated anxiety level. Therefore, we believe that the activation of inflammation may play the key role in the association with anxiety in heart failure patients.

In the present study, we noticed that the distribution of NYHA classification in heart failure patients with or without anxiety was significantly different: majority of patients (91%) with anxiety had NYHA classification of class III and IV, only 70.1% patients without anxiety had NYHA classification of class III and IV. We also have documented the BNP is associated with anxiety in heart failure patients and the LVEF was significantly and negatively related with HAM-A_14_ score. As important reflectors of cardiac function, NYHA classification, BNP and LVEF are widely used factors in predicting the existence and severity of heart failure [33–35]. It was relatively easy to conceive that the anxiety status would significantly increase as the symptoms of heart failure were getting worse.

We found the BUN was significantly associated with anxiety of herat failure patients (*r* = 0.223, *P* < 0.001). The BUN is the indictor for kidney function, the reason for its increase may be as follows. When the cardiac function decreased to a certain level, out body would redistribute the body flow to guarantee the perfusion of vital organ such as heart and brain, the perfusion of kidney might be significantly reduced, the under-perfusion of kidney may cause the accumulation of BUN in the blood [36, 37].BUN alone predicts the prognosis of patients with HF that is influenced by many factors, and BUN / Cr may be more stable and more accurately evaluated than blood creatinine or BUN alone [38].

Interestingly, we have found that female patients were more easily to develop anxiety than male patients, our data showed that the female patients have 3.398 times probability of suffering anxiety than male patients (*P* < 0.001). The sex difference in anxiety in heart failure was rarely documented, however, in population based epidemic studies, the results showed that the lifetime prevalence is higher in women than in men [39–41], women are two to three times more likely than men to have higher self-reported anxiety scores [42].

Our study has several strengths. First, it is the first time the association with NEUT% and anxiety in Chinese hospitalized heart failure patients was discovered. Second, the demographic, clinical and laboratory data in heart failure patients with or without anxiety was documented, which conveyed valuable information on following studies to screen new risk factors for anxiety in Chinese hospitalized heart failure patients. Fourth, NEUT% was contained in the blood routine test, which is simple and cheap to test, NEUT% may provide new insight into the anxiety level in hospitalized heart failure patients. Our study has several limitations.This study was a cross-sectional and observational study, the causal relationship between NEUT% and anxiety need to be further clarified in the following clinical trials. The study was intended to search for confounding factors of anxiety of heart failure patients,. Third, we did not find heart failure patients with severe anxiety in this presented study, more patients would be needed to detect the heart failure patients with severe anxiety.

## Conclusions

In conclusion, Neutrophilic granulocyte percentage is associated with anxiety in Chinese hospitalized heart failure patients. The NEUT% was found to increase with anxiety level categories in Chinese heart failure patients. The NEUT% could be used as a new marker for anxiety in Chinese hospitalized heart failure patients. The cheap and easily acquired indictor could provide us with new insight into the anxiety level in Chinese heart failure patients.

## Data Availability

The datasets generated and analyzed during the present study are available from the corresponding author on reasonable request.
